# Detection of viral respiratory pathogens in mild and severe acute respiratory infections in Singapore

**DOI:** 10.1038/srep42963

**Published:** 2017-02-20

**Authors:** Lili Jiang, Vernon Jian Ming Lee, Lin Cui, Raymond Lin, Chyi Lin Tan, Linda Wei Lin Tan, Wei-yen Lim, Yee-Sin Leo, Louie Low, Martin Hibberd, Mark I-Cheng Chen

**Affiliations:** 1 Saw Swee Hock School of Public Health, National University Health System, National University of Singapore, Singapore; 2 Biodefence Centre, Singapore Armed Forces, Singapore; 3 National Public Health Laboratory, Ministry of Health, Singapore; 4 Department of Microbiology and Immunology, Yong Loo Lin School of Medicine, National University Health System, National University of Singapore, Singapore; 5 Department of Infectious Diseases, Communicable Disease Centre, Tan Tock Seng Hospital, Singapore; 6 Genome Institute Singapore, Singapore; 7 Department of Clinical Epidemiology, Communicable Disease Centre, Tan Tock Seng Hospital, Singapore

## Abstract

To investigate the performance of laboratory methods and clinical case definitions in detecting the viral pathogens for acute respiratory infections (ARIs) from a prospective community cohort and hospital inpatients, nasopharyngeal swabs from cohort members reporting ARIs (community-ARI) and inpatients admitted with ARIs (inpatient-ARI) were tested by Singleplex Real Time-Polymerase Chain Reaction (SRT-PCR), multiplex RT-PCR (MRT-PCR) and pathogen-chip system (PathChip) between April 2012 and December 2013. Community-ARI and inpatient-ARI was also combined with mild and severe cases of influenza from a historical prospective study as mild-ARI and severe-ARI respectively to evaluate the performance of clinical case definitions. We analysed 130 community-ARI and 140 inpatient-ARI episodes (5 inpatient-ARI excluded because multiple pathogens were detected), involving 138 and 207 samples respectively. Detection by PCR declined with days post-onset for influenza virus; decrease was faster for community-ARI than for inpatient-ARI. No such patterns were observed for non-influenza respiratory virus infections. PathChip added substantially to viruses detected for community-ARI only. Clinical case definitions discriminated influenza from other mild-ARI but performed poorly for severe-ARI and for older participants. Rational strategies for diagnosis and surveillance of influenza and other respiratory virus must acknowledge the differences between ARIs presenting in community and hospital settings.

Influenza and other respiratory viruses such as respiratory syncytial virus, rhinovirus, parainfluenza virus, adenovirus and human metapneumovirus are common causes of respiratory infections^1^. While often manifesting as a mild illness, these viruses can result in serious complications^2^,^3^, hospitalisations and deaths^4^.

Identifying the viral aetiology of respiratory infections has applications in both clinical management and surveillance. Early diagnosis may allow timely initiation of appropriate treatment^5^,^6^, and where warranted, rapid confirmation of an outbreak can lend itself to control and mitigation efforts for influenza^7^,^8^. And while specific therapeutic or preventive measures for most viral respiratory agents (other than influenza) are lacking, diagnosis can still help in ruling out other causes of respiratory illness and facilitate implementation of appropriate infection control measures in healthcare settings^9^. Moreover, with increasing concerns about the spread of new and dangerous viral respiratory pathogens such as severe acute respiratory syndrome (SARS) and middle East respiratory syndrome (MERS) viruses, we need to better appreciate how we can optimally combine clinical information with routine as well more complex and expensive laboratory testing technologies to diagnose and conduct surveillance for unusual pathogens^10^.

Current approaches to diagnosis and surveillance rely heavily on clinical case definitions and a variety of laboratory assays. However, overlapping symptoms makes existing clinical case definitions for respiratory infections inadequate for diagnosis of specific infections^11^,^12^. Reverse transcriptase-polymerase chain reaction (RT-PCR), which detects viral nucleic acid by use of amplification techniques, is now considered as the gold standard assay for detection of respiratory viruses, and also has the advantage of short turn-around times as compared to methods based on virus culture and isolation^13^. Moreover, multiplex RT-PCR (MRT-PCR) assays, which allow for rapid detection of multiple types of known viral agents, are now widely available^14^. Studies have evaluated the performance of PCR-based assays for diagnosing respiratory viruses^15^,^16^,^17^, but were mostly restricted to either outpatients or inpatients, or to at-risk groups such as the elderly and young children. Moreover, large-scale pathogen detection technologies, like the Genome Institute of Singapore (GIS) PathChip, have recently been developed^18^, and there is also a need to rationalize how these could be integrated with routinely available assays.

Our study had two key objectives. Firstly, we investigated the performance of the singleplex RT-PCR (SRT-PCR) and MRT-PCR, as well as explored the use of the GIS PathChip for the detection of viral respiratory pathogens for mild and severe acute respiratory illness (ARI). Secondly, we evaluated how well clinical case definitions might differentiate influenza from other causes for mild and severe ARIs. We collected samples and clinical data from both a community cohort and adult inpatients to address the first objective while we combined this with data from mild and severe influenza cases prospectively recruited during the influenza A(H1N1)pdm09 pandemic of 2009 to support the second objective.

## Results

Of 507 participants from the community enrolled into a cohort, 99 reported having ARI episodes. We also enrolled 142 inpatients from Tan Tock Seng Hospital (TTSH), and retrieved 128 influenza cases from a previous study (historical-FLU). Community participants with ARI episodes did not differ significantly from the underlying cohort (Table 1). Compared to community participants with ARI, inpatients were older, more likely to be male and to have chronic medical conditions. Similarly, historical-FLU participants with medical indications were older, and more likely to have chronic medical conditions as compared to those who were admitted for public health indications. Historical-FLU participants with medical indications were fairly similar to the inpatients although they were slightly younger and less likely to have COPD and heart disease.

One hundred and thirty episodes were reported from 99 community participants (community-ARI) with 76, 16, 6 and 1 individuals reporting 1, 2, 3 and 4 episodes respectively. For the inpatients, there were 145 episodes with only 1 individual having 2 and another having 3 episodes (inpatient-ARI).

### Viruses detected by SRT-PCR and MRT-PCR in Community-ARI and Inpatient-ARI episodes

On using SRT-PCR with MRT-PCR, 53.8% of the community-ARI episodes were diagnosed as panel virus positive (i.e. positive for at least one of the viruses on the MRT-PCR panel used), including 10 (7.7%) influenza virus positive episodes (8A(H3N2), 1A(H1N1)pdm09 and 1 influenza B) and 60 (46.2%) non-influenza panel virus positive episodes. Two viruses were more common than influenza in community-ARI, with 37 (28.5%) episodes positive for rhinoviruses and 13 (10.0%) for coronaviruses (Table 2). There were no co-infections detected by MRT-PCR in either community-ARI episodes or influenza negative inpatient-ARI. However, 5 influenza positive inpatient-ARI episodes were positive for more than one pathogen, including 4 dual-pathogen (influenza A + influenza B [2], influenza A + rhinovirus, and influenza B + respiratory syncytial virus) and 1 triple-pathogen (influenza A + adenovirus + Coronavirus) episodes. Another 46 inpatient-ARI episodes were positive only for one influenza type/subtype; 23(50.0%), 9(19.6%) and 14(30.4%) of these episodes were influenza A(H3N2), influenza A(H1N1)pdm09 and influenza B respectively. After excluding episodes with multiple pathogens, only 24 inpatient-ARI episodes were positive for non-influenza panel viruses, with 8 (33.3%) and 7 (29.2%) of these being rhinoviruses and coronaviruses respectively; another 70 episodes were negative for all panel viruses. The ratio of influenza negative to influenza positive episodes was 1.8:1, which reflected the intention of our study design to sample non-influenza to influenza episodes in an approximately 2:1 ratio. However, in routinely ordered diagnostic tests for influenza conducted on 6281 TTSH admission episodes during the study period, 11.0% tested positive (354A(H3N2), 118A(H1N1)pdm09, 176 influenza B and 40 influenza A with undefined subtype detected). Using this to adjust for the effect of our inpatient-ARI sampling strategy, we estimated that only 18.0% of inpatient-ARI were positive for a non-influenza virus, and an estimated 71.0% would be negative for all panel viruses, as compared to 46.2% for community-ARI.

### Comparison of SRT-PCR versus MRT-PCR and additional viruses detected by PathChip in community-ARI and inpatient-ARI episodes

Of the 138 and 207 samples from community-ARI and inpatient-ARI episodes, 18 and 113 respectively were for influenza positive episodes (where multiple samples were collected for each episode). The time between episode onset and the first sample for inpatient-ARI was longer than for community-ARI (median of 5 days vs 1 day respectively, p < 0.001); however, no such difference was observed between influenza versus other ARI episodes either for community-ARI or inpatient-ARI (p = 0.387 and p = 0.481 respectively).

**Figure 1:**
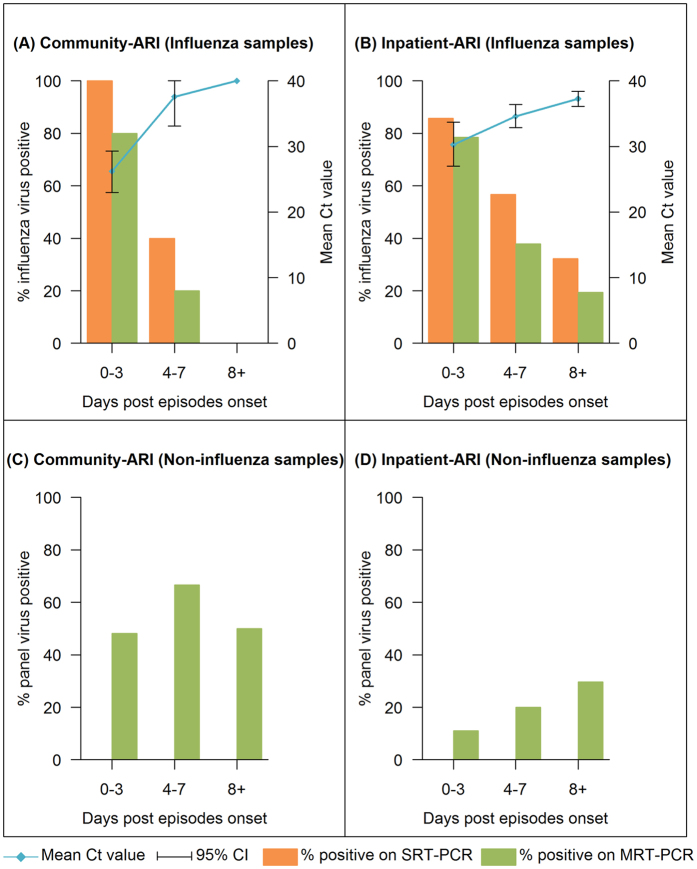
Comparison of virus detection by days post-onset using SRT-PCR and MRT-PCR. Detection of influenza in influenza-related samples from community-ARI (**A**) and inpatient-ARI (**B**), and non-influenza virus detection in all samples from community-ARI (**C**) and inpatient-ARI (**D**). Confidence intervals (CIs) were provided for Ct values.

We compared the detection of influenza and non-influenza panel viruses by days post-onset. MRT-PCR detected influenza in only 70.8% of the samples that were positive for influenza by SRT-PCR. For influenza positive episodes, the proportion positive by either assay decreased as days post-onset increased for both the community and inpatient samples (Fig. 1A,B). The SRT-PCR Ct values also increased with days post-onset in both groups, but the Ct value for community samples increased more sharply than for the inpatients. The GEE model suggested that inpatient-ARI had a higher starting Ct value than community-ARI (P = 0.002, Table 3). Ct value increased as days post-onset increased (P < 0.001), but the increase in Ct value with every additional day post-onset for inpatient-ARI was lesser than that for community-ARI (P < 0.001 for interaction term between participant type and days post episode onset). For non-influenza panel virus episodes, changes in the proportion of positive samples showed no consistent pattern by days post-onset in both community-ARI and inpatient-ARI (Fig. 1C,D).

Amongst 131(No samples were sent for PathChip analysis for 4 PCR negative inpatient-ARI episodes) PCR negative episodes, PathChip did not detect any viruses in 98 episodes. In 6 episodes, multiple viruses (both respiratory and non-respiratory) were detected; these were excluded from further analysis. 10 episodes were positive to MRT-PCR panel virus; all were from community-ARI, with 8 positive for rhinovirus, 1 for influenza A and another 1 for metapneumovirus. 13 more episodes (11 community-ARI, 2 inpatient-ARI) were positive for viruses including coxsackievirus (1), human enterovirus(9), human parainfluenza virus 4 (1) and influenza C virus (2) where ARI is a recognised presentation^19^. 4 episodes had only non-ARI viruses detected (Human herpesvirus 5, Human papillomavirus, Molluscum contagiosum virus and Human T-lymphotropic virus 2). The PathChip results increased the proportion of community-ARI episodes positive for respiratory viral pathogens from 53.8% to 70.0%, and from 63.8% to 78.7% for episodes meeting febrile respiratory illness (FRI) criteria (i.e. ARI with self-reported fever, regardless of body temperature measurement).

### Detection of influenza and other viruses using clinical criteria on community-ARI, inpatient-ARI and historical-FLU episodes grouped as mild and severe ARI

Due to the small number of influenza positive episodes in community-ARI, we combined community-ARI and inpatient-ARI episodes with influenza episodes identified from a previous study (described further in Supplementary Material). Clinical features of historical-FLU admitted for public health indications were fairly similar to influenza cases identified amongst community-ARI, which justified grouping them together as mild-ARI (i.e. community-ARI + historical-FLU, public health indications); likewise historical-FLU with medical indications for admission was fairly similar to influenza from inpatient-ARI, and were designated severe ARI (i.e. inpatient-ARI + historical-FLU, medical indications, also see Supplementary Table S1). None of the respiratory symptoms assessed were significantly more common in influenza, and for mild-ARI, sore throat and runny nose were significantly less common in those testing positive for influenza (Table 4). However, for both mild and severe ARI, FRI and influenza-like illness (ILI) case definitions as well as temperature cut-off points showed significant discriminatory value for influenza over non-influenza episodes or any panel viruses positive over viruses negative episodes.

**Figure 2:**
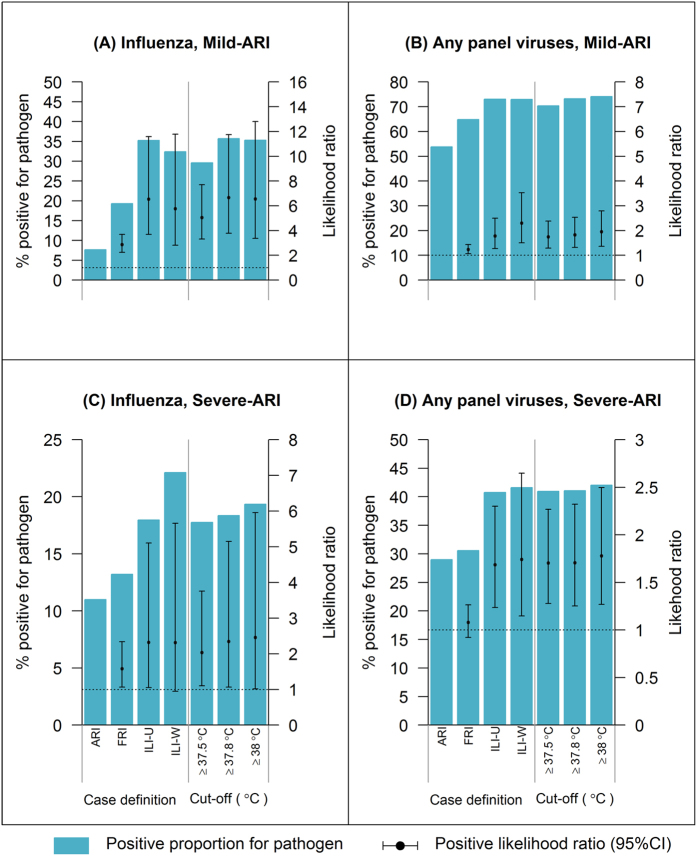
Comparison of the ability of different case definitions and temperature cut-off points in discriminating influenza, other viral respiratory infections and ARIs negative on MRT-PCR. Discriminating influenza from other causes of ARI in mild-ARI (**A**) and severe-ARI (**C**), and discriminating infections positive for any MRT-PCR panel viruses from samples negative by MRT-PCR for mild-ARI (**B**) and severe-ARI (**D**). Figure does not include additional positive results from the PathChip system.

Figure 2A shows that in mild-ARI, while only 7.7% was influenza positive by SRT-PCR, this rose to 19.3%, 35.2% and 32.4% for FRI, ILI-U (US Centers for Disease Control ILI definition) and ILI-W (World Health Organisation ILI definition) respectively, with LR+ >5 for ILI case definitions and temperature cut-off points ≥37.8 °C. While using ILI-W increased the proportion positive for any MRT-PCR panel viruses to 73.0%, this represented a relatively small improvement over the 53.8% positive in all mild-ARI, with LR+ of only 2.3 (Fig. 2B). For severe-ARI, use of more specific case definitions also improved the likelihood of being positive for influenza and viral respiratory infection, but the LR+ only ranged from 1 to 2.5. Notably, for severe-ARI, ILI-W performed best, with 22.1% and 41.6% of such episodes positive for influenza and any panel viruses respectively (Fig. 2C,D). We performed a sensitivity analysis excluding the historical-FLU cases and the results were essentially the same other than for the wider confidence intervals (due to the reduced sample size, see Supplementary Fig. S1).

There were insufficient numbers of mild-ARI with age ≥60 years for age-stratified analysis, but influenza was significantly more likely than non-influenza episodes to meet FRI, ILI-U and ILI-W criteria for both mild-ARI and severe-ARI in those aged <60 years (Table 5). However, case definitions had poorer discriminatory value in severe-ARI and older ages. For instance, in those aged <60 years, severe influenza was significantly more likely than mild influenza to meet ILI-W criteria (68.6% vs 48.6% respectively, p = 0.018), but non-influenza causes of severe-ARI were also more likely to have febrile presentations, with 32.6% meeting ILI-W criteria (vs 8.9% for non-influenza mild-ARI, p < 0.001). In addition, comparing severe influenza between age groups shows that episodes in older individuals were also significantly less likely than to meet ILI criteria (e.g. for ILI-U, 77.1% for severe-ARI in those aged <60 years versus only 51.3% for those aged ≥60 years, p = 0.010).

## Discussion

Our study concurrently assessed the role of routine laboratory diagnostics, and usefulness of the novel PathChip platform as well as ILI case definitions in identifying respiratory virus infection in a community cohort and hospital inpatients from a broad range of age groups (6 to 81, and 20 to 89 years respectively), to reflect what may be encountered in either community or primary care (mild-ARI) as well as tertiary care settings (severe-ARI) in a tropical environment with less distinct seasonal patterns.

Our study clarifies the role of singleplex and multiplex RT-PCR in the respective populations. We observed imperfect sensitivity of MRT-PCR for influenza (70.8%) as compared to SRT-PCR, but better performance was reported in two outpatient studies (91–96%)^15^,^20^, possibly related to the longer time between onset and sample collection for inpatients in our study, as the recovery of samples positive for influenza and sensitivity of MRT-PCR for influenza was dependent on the time between onset and sample collection. Post episode onset, the proportion positive for influenza decreased (Ct value increased) faster in community-ARI as compared to inpatient-ARI, but no such pattern was observed for non-influenza panel viruses; likewise, others have found slower viral clearance in inpatients compared to outpatients for influenza A(H1N1)pdm09^21^, and a lack of association between days post-onset and Ct values for adenovirus, human metapneumovirus, parainfluenza virus 1–3^22^. PCR-based assays may thus retain greater value for detecting influenza amongst inpatients that present late than in the community where episodes presenting more than 7 days post-onset would likely be influenza negative.

Our study also explored the application of newer platforms like the PathChip for detecting respiratory virus infections. In contrast to multiplexed PCR-based technologies which rely on a set of primers that target a finite number of specific pathogens, the PathChip is designed to detect a wide array of pathogens simultaneously by detecting signatures in the pathogen genome sequences. While costly, our results suggests that it has the potential to complement existing technologies as a diagnostic tool. Simões et al previously evaluated the diagnostic value of the PathChip using paediatric nasal wash samples and reported variable sensitivity ranging from 30.8% to 95.0% and reasonable specificity from 88.4% to 100%^18^. In our study, the PathChip was a valuable addition in community-ARI where it substantially increased the proportion positive for respiratory etiological agents, so that only about 20% of febrile episodes did not have a viral aetiology identified. Notably, the vast majority of additional viruses detected were those known to cause ARI. It may thus help in ruling out more sinister causes of ARI if added to MRT-PCR for surveillance in community settings; it may also help detect unexpected but dangerous infections such as SARS and MERS-CoV following further validation on actual patient samples with such infections. However, due to the high cost of the assay (about four times MRT-PCR) and the imperfect sensitivity for some pathogens (as low as 30.8% for adenovirus), we opted to test only the RT-PCR negative samples with the PathChip. As such, we are unable to comment on its sensitivity for samples from adults, which may be inferior to the previously published results which used samples from paediatric subjects, who are known to have higher viral loads for some viruses^23^,^24^. However, given its current cost, variable sensitivity and slower turn-around time as compared to MRT-PCR (about 20 hours for PathChip), we believe this technology is most appropriately used in the way we designed our study, which is to detect additional respiratory viral pathogen positive episodes as an adjunct to more widely available MRT-PCR panels designed to cover the most common pathogens from community-based samples.

Finally, our study highlights how the same clinical case definitions, which have been used in both community^11^ and inpatient settings^25^, may actually perform differently in the two settings for distinguishing influenza from other respiratory infections, identifying influenza cases, and increasing the yield of diagnostic assays. We found that, while respiratory symptoms themselves are poor in distinguishing influenza from other ARI causes, clinical case definitions using fever or temperature cut-off points demonstrated good discriminatory value in our mild-ARI episodes, which should reflect what can be anticipated in primary care settings. However, for ARI severe enough to require hospitalisation, the case definitions had poorer discriminatory value. This was partly because, while a good majority of older individuals (87.2%) with severe influenza had self-reported fever (as in FRI), only about half met the temperature criteria used for ILI case definitions (Table 5). The use of such high temperature cut-off points would hence substantially reduce sensitivity for detecting influenza in the elderly. Also, non-influenza episodes in ARI severe enough to require hospitalization are more likely to also have a higher temperature than non-influenza episodes in mild ARI; this further reduces the ability of ILI case definitions to distinguish influenza from other respiratory causes in the inpatient setting. The choice of an appropriate case definition hence depends on the objectives and setting of the application. If the aim is to diagnose as many influenza cases as possible (e.g. for identifying patients for antiviral treatment or outbreak management), ILI case definitions have inadequate sensitivity, particularly in older age groups. However, the discriminatory value of ILI criteria finds application when aiming to reduce background noise during syndromic surveillance, either in community type settings or even a healthcare worker population^26^. The ILI case definitions are also useful for optimizing the yield of influenza viruses from samples tested, and particularly efficient in settings where milder ARI presentations are encountered (Fig. 2A); and if the aim is to identify all types of respiratory viruses for surveillance, ILI criteria would also modestly improve the probability of obtaining a positive result as compared to sampling all ARI in both mild as well as severe presentations (Fig. 2B,D).

A key limitation we acknowledge is the less than ideal number of influenza infections identified through our community cohort, which led us to supplement our concurrent community and inpatient studies with data from our older prospective study of influenza cases. While not ideal, Supplementary Table S1 suggests that influenza cases from this historical dataset which were classified as mild were reasonably similar to those from the community cohort, and likewise those classified as severe were similar to those from the later inpatient study. A sensitivity analysis without the influenza cases from our historical dataset gave a similar result. However, even with these additional influenza cases, the numbers remained inadequate to assess the performance of the case definitions for mild influenza in older age groups, and we are also unable to assess performance in paediatric subjects. Secondly, the panel of viruses tested on the multiplex PCR assay was not comprehensive; while this was supplemented by the PathChip, there is no means of ascertaining what proportion of the remaining ARI episodes are truly not due to an infectious viral aetiology. We also recognise that our study population, in particular the community cohort, may not be representative of the general community, which was further complicated by fluctuations in the rate at which participants notified us of ARI episodes, as well as intra-seasonal variations within each year and sporadic outbreaks of particular respiratory pathogens^27^.

## Conclusion

The performance of various technologies and case definitions for viral respiratory pathogens presenting with ARI differs substantially between community and hospital-based settings. PCR-based assays may still be relevant for detecting influenza amongst inpatients that present late but less so for community ARI episodes with delayed presentations. The PathChip may add value for respiratory virus detection in samples negative by multiplex RT-PCR, but only for community-ARI. Finally, US-CDC and WHO ILI case definitions had similar performance for mild ARI, but performed less adequately amongst presentations severe enough to require hospitalisation, including in older individuals. Rational strategies for diagnosis and surveillance of influenza and other respiratory viruses must acknowledge the differences between these two populations.

## Methods

### Study population

We recruited a community cohort and inpatients from TTSH between April 2012 and December 2013.

The community cohort was recruited by contacting community-dwelling adults who participated in pre-existing prospective cohort studies conducted by the National University of Singapore (NUS) Saw Swee Hock School of Public Health. Consenting individuals were then enrolled through home visits, where we also opportunistically recruited other members of the household, including children. At enrolment, participants contributed demographic and health information through a baseline interview at enrolment, and then followed-up for up to 1.5 years, with instructions to notify the study team within 24 hours on developing ARI symptoms (community-ARI). Once notified, research staff would obtain nasopharyngeal swabs and symptom data from the participant on the next working day.

For inpatients, on each working day, we would screen through the list of adults admitted to TTSH within the last 72hrs who had undergone routine diagnostic testing (by SRT-PCR) for influenza. We then enrolled and collected nasopharyngeal swabs from consenting inpatients fulfilling the same ARI criteria (inpatient-ARI) used for the community cohort. To better characterise influenza, we intentionally oversampled influenza to obtain a ratio of approximately 1 influenza positive to 2 influenza negative patients.

Additional swabs were taken on days 3 to 5 and days 7 to 9 post-onset (community-ARI) or post-enrolment (inpatient-ARI) for influenza positive episodes where possible.

In addition, we retrieved historical influenza data from a prospective study of admissions to TTSH testing positive for influenza between May 2009 and September 2009 (historical-FLU). This included ARI patients with medical indications who were admitted alongside ARI cases referred to TTSH for public health indications (clinically suspected to have influenza A(H1N1)pdm09 based on epidemiological risk factors like travel and contact history).

Ethics approvals were obtained from NUS Institutional Review Board and the National Healthcare Group (Singapore) Domain Specific Review Board, in accordance with relevant guidelines and regulations. A written informed consent was signed by each study participant.

### Laboratory analysis

Three assays were performed. SRT-PCR and MRT-PCR assays were conducted at the NPHL on all available samples while PathChip assays were performed at GIS using RT-PCR negative samples.

### SRT-PCR and MRT-PCR

Nasopharyngeal swabs (Copan, Italy) were transported in RT-UTM (Copan, Italy) to the National Public Health Laboratory (NPHL), where nucleic acids were extracted by using EZ1 Virus Mini Kit (Qiagen, Cat No. 95514).

#### SRT-PCR

This assay was only used to detect influenza virus types A and B at a higher sensitivity than might be achieved using multiplexed PCR assays, and to further subtype influenza A positive samples. The protocols had been adopted from the studies by Spackman *et al*.^28^ and Krafft *et al*.^29^ and, respectively, with cycle threshold (Ct) values documented for positive samples (samples with a Ct value less than 40 were considered as positive). Influenza A positive specimens were then subtyped with subtype specific primers and probes that targeted at haemagglutinin (HA) gene of A(H3N2)3, and HA and nucleoprotein (NP) genes of A(H1N1)pdm09^30^. Cycle threshold (Ct) values were documented for positive samples.

#### MRT-PCR

This assay was used to simultaneously detect multiple common respiratory viruses. The SeeGene RV12 kit was used according to the manufacturer’s instructions. The panel included 12 viruses: influenza A, influenza B, respiratory syncytial virus A, respiratory syncytial virus B, parainfluenza virus 1, parainfluenza virus 2, parainfluenza virus 3, human metapneumovirus, rhinovirus A/B, adenovirus A/B/C/D/E, human coronaviruses 229E/NL63 and human coronaviruses OC43(referred to henceforth as panel virus).

#### PathChip

This assay was used to detect additional viruses beyond what was covered by the primers within the MRT-PCR panel used. For each sample, the cDNA was amplified from extracted nucleic acid. The novel platform from the Genome Institute of Singapore (GIS) then automatically detects which pathogens’ recognition signatures are present, based on a proprietary algorithm constructed based on genetic sequences of viruses clinically relevant to humans (downloaded from the NCBI Taxonomy data-base, http://www.ncbi.nlm.nih.gov/Taxonomy/taxonomyhome.html/)^18^.

### Statistical analysis

We compared the ability of SRT-PCR and MRT-PCR to detect influenza in terms of days post-onset and Ct values (generated from SRT-PCR for influenza samples only). A generalized estimating equation (GEE) model was applied to evaluate the effect of study population and days post-onset on the Ct values. Only samples collected before the initiation of antiviral treatment were included.

To evaluate how the GIS-PathChip might add to detection above routinely available MRT-PCR assays, viruses were grouped into panel viruses (i.e. virus group represented in SeeGene RV12), non-panel viruses for which ARI is a common presentation^19^ and non-ARI viruses.

In our analysis on the combined performance of clinical case definitions and laboratory assays, we grouped all community-ARI episodes (none of which required hospitalisation) with historical-FLU admitted for public health indications; these were designated as “mild-ARI” since these would ordinarily not be sufficiently severe as to require hospitalisation. Inpatient-ARI was grouped with historical-FLU cases admitted for medical indications (“severe-ARI”). This increased the number of influenza cases available for evaluating the discriminatory value of clinical parameters and case definitions, which were:

- ARI: episode with acute onset with any key respiratory symptoms including cough, shortness of breath, sore throat, or runny nose.
- Febrile respiratory illness (FRI): ARI with self-reported fever, regardless of body temperature (T) measurement.
- Influenza-like illness defined by Centers for Disease Control and Prevention of the United States of America (ILI-U): fever ≥37.8 °C together with cough and ⁄or sore throat in the absence of a known cause other than influenza^31^.
- Influenza-like illness defined by World Health Organization (ILI-W): fever of ≥38 °C plus cough with onset within the last 10 days^32^.

The proportion of episodes fulfilling different criteria was compared by viral pathogen status (influenza viruses, non-influenza panel viruses and negative), and we then investigated the discriminatory performance by calculating the positive likelihood ratio (LR+) and its 95% confidence intervals (CI) for positive detection of influenza and any viral infection. This was also done to investigate the effect of age (age <60 vs ≥60 years). Estimates of LR+ requires assumptions on the anticipated prevalence of influenza. For community-ARI, this was assumed to be what was obtained in ARI samples from the community cohort. For inpatient-ARI, since influenza positive inpatients were intentionally oversampled (Crude_FLU), the proportion positive for influenza was estimated based on the proportion positive for influenza from routinely ordered tests of ARI admissions to TTSH (Adjusted_FLU), which was then applied to derive the adjusted (Adjusted_Non-FLU) from the crude proportion (Crude_Non-FLU) positive for non-influenza viruses detected in MRT-PCR using the following formula:


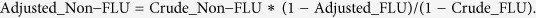


All analyses were conducted using R version 3.0.1 (R Foundation for Statistical Computing, Vienna, Austria).

## Additional Information

**How to cite this article**: Jiang, L. *et al*. Detection of viral respiratory pathogens in mild and severe acute respiratory infections in Singapore. *Sci. Rep.* **7**, 42963; doi: 10.1038/srep42963 (2017).

**Publisher's note:** Springer Nature remains neutral with regard to jurisdictional claims in published maps and institutional affiliations.

## Supplementary Material

Supplementary Material

## Figures and Tables

**Table 1:** Comparison of baseline characteristics of community cohort participants, community participants with ARI^a^, inpatients, and Historical-FLU^b^ with public health and medical indications for admission.

	Community cohort (n = 507)	Community cohort with ARI (n = 99)	Inpatients (n = 142)	Historical-FLU, public health indications^c^ (n = 65)	Historical-FLU, medical indications^d^ (n = 63)
Age in years					
Median (IQR)	43 (25–52)	39 (21–50)	61 (52–67)	32 (26–40)	41 (25–53)
Age group (%)					
<20	97 (19.1)	24 (24.5)	0 (0.0)	0 (0.0)	0 (0.0)
20–39	129 (25.4)	27 (27.5)	14 (9.9)	48 (73.9)	28 (44.4)
40–59	233 (46.0)	44 (42.9)	51 (35.9)	16 (24.6)	24 (38.1)
60+	48 (9.5)	4 (5.1)	77 (54.2)	1 (1.5)	11 (17.5)
Gender (%)					
Female	295 (58.4)	59 (59.6)	47 (33.1)	25 (38.5)	33 (52.4)
Medical conditions (%)					
Diabetes	52 (10.3)	7 (7.1)	30 (21.1)	0 (0.0)	16 (25.4)
Asthma	33 (6.5)	7 (7.1)	36 (25.4)	2 (3.1)	20 (31.8)
COPD	3 (0.6)	1 (1.0)	19 (13.4)	1 (1.5)	1 (1.6)
Heart disease	8 (1.6)	0 (0.0)	26 (18.3)	1 (1.5)	4 (6.4)
Others	10 (2.0)	2 (2.0)	43 (30.3)	3 (4.6)	6 (9.5)

^a^ARI: episode with acute onset with any key respiratory symptoms including cough, shortness of breath, sore throat, or runny nose.

^b^Historical-FLU: historical influenza data from a prospective study of admissions to TTSH testing positive for influenza between May 2009 and September 2009 (historical-FLU).

^c^Historical-FLU, public health indications: ARI cases referred to TTSH for public health indications following clinical suspicion of having influenza A(H1N1)pdm09 based on epidemiological risk factors like travel and contact history.

^d^Historical-FLU, medical indications: patients with medical indications requiring hospitalisation for care and treatment.

**Table 2:** Non-influenza mono-infection episodes in community-ARI and inpatient-ARI episodesVirus identified.

	Community-ARI			Inpatient-ARI^c^	
	No. of episodes	% of all episodes^a^	% of non-influenza positive episodes^b^	No. of episodes	% of non-influenza negative episodes^d^
Adenovirus	1	0.8	1.7	1	5.3
Human coronaviruses^e^	13	10.0	21.7	7	36.8
Human metapneumoviruses	1	0.8	1.7	0	0.0
Parainfluenza viruses	3	2.3	5.0	1	5.3
Respiratory syncytial viruses	5	3.8	8.3	2	10.5
Rhinoviruses	37	28.5	61.7	8	42.1

^a^As proportion of all 130 Community-ARI episodes.

^b^As proportion of 60 Community-ARI episodes positive for non-influenza viruses.

^c^For inpatient-ARI, this excludes 5 episodes where there was a co-infection between influenza and non-influenza viruses on MRT-PCR.

^d^As proportion of 24 Inpatient-ARI mono-infection episodes positive for non-influenza viruses.

^e^Includes HCoV 229E/NL63 and HCoV OC43.

**Table 3:** Outcome of the GEE model^a^ evaluating the factors on the dynamics of Ct values.

Factors	Coefficient	95% CI	p-value
Inpatients (versus community)	7.07	2.60, 11.50	0.002
Days post episode onset stratified by participant type			
Community	2.06	1.68, 2.45	<0.001
Inpatients	0.58	0.32, 0.83	<0.001

^a^Generalized Estimating Equation model which included participant type (community cohort or inpatient), days post episode onset, age, gender, comorbidities (comparing those who reported any comorbidities with those who didn’t report any comorbidities, with the comorbidities included being diabetes, asthma, COPD, heart disease, cancer and other significant conditions), as well as the interaction term between participant type and days post episode onset.

**Table 4:** Clinical criteria and case definitions by PCR assay result for mild-ARI and severe-ARI.

	Symptoms/Case definitions	Total	Viruses^a^			P value
			Influenza viruses (%)	Non-influenza viruses (%)	Negative (%)	
Mild-ARI^b^		N = 195	n = 75	n = 60	n = 60	
	Cough	170 (87.2)	67 (89.3)	54 (90.0)	49 (81.7)	0.347
	Sore throat	150 (76.9)	50 (66.7)	51 (85.0)	49 (81.7)	0.027
	Breathlessness	18 (9.2)	5 (6.7)	9 (15.0)	4 (6.7)	0.216
	Runny nose	149 (76.4)	41 (54.7)	55 (91.7)	53(88.3)	<0.001
	T ≥ 37.5 °C	79 (40.5)	60 (80.0)	11 (18.3)	8 (13.3)	<0.001
	T ≥ 37.8 °C	62 (31.8)	50 (66.7)	7 (11.7)	5 (8.3)	<0.001
	T ≥ 38.0 °C	51 (26.2)	41 (54.7)	6 (10.0)	4 (6.7)	<0.001
	FRI^d^	109 (55.9)	70 (93.3)	22 (36.7)	17 (28.3)	<0.001
	ILI-U^e^	61 (31.3)	49 (65.3)	7 (11.7)	5 (8.3)	<0.001
	ILI-W^f^	46 (23.6)	36 (48.0)	6 (10.0)	4 (6.7)	<0.001
Severe-ARI^c^		N = 203	n = 109	n = 19	n = 75	
	Cough	200 (98.5)	107 (98.2)	19 (100.0)	74 (98.7)	1.000
	Sore throat	98 (48.3)	52 (47.7)	11 (57.9)	35 (46.7)	0.711
	Breathlessness	105 (51.7)	50 (45.9)	11 (57.9)	44 (58.7)	0.220
	Runny nose	113 (55.7)	55 (50.5)	12 (63.2)	46 (61.3)	0.287
	T ≥ 37.5 °C	118 (58.1)	79 (72.5)	11 (57.9)	28 (37.3)	<0.001
	T ≥ 37.8 °C	112 (55.2)	76 (69.7)	10 (52.6)	26 (34.7)	<0.001
	T ≥ 38.0 °C	104 (51.2)	72 (66.1)	9 (47.4)	23 (30.7)	<0.001
	FRI	170 (83.7)	100 (91.7)	14 (73.7)	56 (74.7)	0.003
	ILI-U	110 (54.2)	74 (67.9)	10 (52.6)	26 (34.7)	<0.001
	ILI-W	88 (43.3)	64 (58.7)	6 (31.6)	18 (24.0)	<0.001

^a^Does not include results from PathChip as this assay is not expected to be routinely available.

^b^Combines the community-ARI and the historical-FLU with public health indications

^c^Combines the inpatient-ARI and the historical-FLU with medical indications

^d^FRI: ARI with self-reported fever, regardless of body temperature (T) measurement

^e^ILI-U: ILI as defined by Centers for Disease Control and Prevention of the United States of America, i.e. fever ≥37.8 °C together with cough and ⁄or sore throat in the absence of a known cause other than influenza.

^f^ILI-W: ILI as defined by World Health Organization, i.e. fever of ≥38 °Cplus cough with onset within the last 10 days.

**Table 5:** Effect of age on the clinical diagnosis for influenza.

Symptoms/Case definitions	Mild-ARI, age < 60 years			Severe-ARI, age < 60 years			Severe-ARI, age ≥ 60 years		
	Influenza	Non-influenza	P value	Influenza	Non-influenza	P value	Influenza	Non-influenza	P value
	n = 74	n = 112	—	n = 70	n = 43	—	n = 39	n = 51	—
Cough	66 (89.2)	98 (87.5)	0.819	68 (97.1)	42 (97.7)	1.000	39 (100)	51 (100)	1.000
Sore throat	49 (66.2)	92 (82.1)	0.015	38 (54.3)	26 (60.5)	0.562	14 (35.9)	20 (39.2)	0.828
Breathlessness	5 (6.8)	13 (11.6)	0.320	29 (41.4)	23 (53.5)	0.246	21 (53.8)	32 (62.7)	0.517
Runny nose	40 (54.1)	100 (89.3)	<0.001	39 (55.7)	30 (69.8)	0.166	16 (41)	28 (54.9)	0.209
T ≥ 37.5 °C	60 (81.1)	19 (17)	<0.001	59 (84.3)	20 (46.5)	<0.001	20 (51.3)	19 (37.3)	0.204
T ≥ 37.8 °C	50 (67.6)	12 (10.7)	<0.001	56 (80)	19 (44.2)	<0.001	20 (51.3)	17 (33.3)	0.130
T ≥ 38.0 °C	41 (55.4)	10 (8.9)	<0.001	54 (77.1)	19 (44.2)	<0.001	18 (46.2)	13 (25.5)	0.047
FRI	69 (93.2)	38 (33.9)	<0.001	66 (94.3)	33 (76.7)	0.008	34 (87.2)	37 (72.5)	0.120
ILI-U	49 (66.2)	12 (10.7)	<0.001	54 (77.1)	19 (44.2)	<0.001	20 (51.3)	17 (33.3)	0.130
ILI-W	36 (48.6)	10 (8.9)	<0.001	48 (68.6)	14 (32.6)	<0.001	16 (41.0)	10 (19.6)	0.035
