# Supplementary material for: Detection of viral respiratory pathogens in mild and severe acute respiratory infections in Singapore
Source: Sci Rep. 2017 Feb 20;7:42963. doi: 10.1038/srep42963 (PMC5317157; doi:10.1038/srep42963)
Supplement: Supplementary Material [file srep42963-s1.doc]

**Detection of viral respiratory pathogens in mild and severe acute respiratory infections in Singapore**

**Lili Jiang ^1^ , Vernon Jian Ming Lee ^1,2^ , Lin Cui ^3^ , Raymond Lin ^3,4^, Chyi Lin Tan ^5^ , Linda Wei Lin Tan ^1^ , Wei-yen Lim ^1^ , Yee-Sin Leo ^5^ , Louie Low ^6^,** [**Martin Hibberd**](http://jcm.asm.org/search?author1=Martin+L.+Hibberd&sortspec=date&submit=Submit) **^6^, Mark I-Cheng Chen ^1,7*^**

1 Saw Swee Hock School of Public Health, National University Health System, National University of Singapore, Singapore, Singapore.

2 Biodefence Centre, Singapore Armed Forces, Singapore, Singapore.

3 National Public Health Laboratory, Ministry of Health, Singapore.

4 Department of Microbiology and Immunology, Yong Loo Lin School of Medicine, National University Health System, National University of Singapore, Singapore, Singapore.

5 Department of Infectious Diseases, Communicable Disease Centre, Tan Tock Seng Hospital,

Singapore, Singapore.

6 Genome Institute Singapore, Singapore, Singapore.

7 Department of Clinical Epidemiology, Communicable Disease Centre, Tan Tock Seng Hospital,

Singapore, Singapore.

****Corresponding author:*** Mark Chen I-Cheng: ephcicm@nus.edu.sg

Saw Swee Hock School of Public Health, National University Health System, National University of Singapore, 12 Science Drive 2, Kent Ridge, 117597, Singapore, Singapore

**Supplementary material**

Study of influenza in acute respiratory infections (ARI) during the influenza A(H1N1pdm09) pandemic

In April 2009, a novel strain of influenza A(H1N1) emerged and spread worldwide quickly. Singapore reported its first imported case on 26 May 2009. Tan Tock Seng Hospital (TTSH) was the designated facility for isolation and treatment of adult patients during the initial phases of the epidemic in Singapore. During the *containment phase* as mandated by the Ministry of Health (from 27 April to 28 June of 2009), all confirmed, probable and suspected influenza A(H1N1)pdm09 cases were referred to TTSH for diagnosis and isolation and treatment; following transition to a mitigation phase (29 June 2009 onwards) only influenza cases that had clinical indications for admission were hospitalised^1^. Most of the earliest cases referred to TTSH were on the basis of travel associated risk factors for infection with influenza A (H1N1) pdm09 ^2^.

A prospective observational study on influenza-related admissions was conducted on patients presenting with ARI symptoms and admitted to TTSH from May 2009 to September 2009, with demographic data, travel-related information, clinical presentations and respiratory samples collected for each consenting participant. Ethics approval for the study was obtained from the National Healthcare Group (Singapore) Domain Specific Review Board.

Given the small number of influenza positive community-ARIs (10 out of 130 ARI episodes), we drew on this data to provide additional corroboration that the influenza positive community-ARI episodes were similar to larger collections of ARIs positive for influenza, and to increase the validity of comparisons made between milder and severe presentations of influenza.

For this purpose, we retrieved historical influenza data from a prospective study of admissions to TTSH testing positive for influenza between May 2009 and September 2009 (historical-FLU). This included ARI patients with medical indications who were admitted alongside ARI cases referred to TTSH for public health indications (clinically suspected to have influenza A(H1N1)pdm09 based on epidemiological risk factors like travel and contact history). In the main manuscript, we grouped community-ARI episodes with historical-FLU admitted for public health indications as “mild-ARI” since these would ordinarily not be sufficiently severe as to require hospitalisation, while inpatient-ARIs were grouped with historical-FLU cases admitted for medical indications (“severe-ARI”). This increased the number of influenza cases available for evaluating the discriminatory value of clinical parameters and case definitions.

Table S1 compares the clinical presentation of influenza in community-ARI and historical-FLU with public health indications. There were no statistically significant differences between these two groups. We also compared the difference between influenza in inpatient-ARIs and historical-FLU with medical indications. Again, there was no statistically significant difference between these two groups in terms of respiratory symptoms. However, historical-FLU with medical indications was more likely to have fever more than 37.5 ^o^C and meet ILI case definitions than influenza in inpatient-ARI.

**Table. S1. Comparison of influenza positive ARIs for the community cohort, inpatients** and the historical study

| Symptoms/  Case definitions | Influenza positive cases from: | | | | | |
| --- | --- | --- | --- | --- | --- | --- |
|  | Community cohort (%) | Historical-FLU, public health indications^2^ (%) | P value | Inpatients (%) | Historical-FLU, medical indications^3^ (%) | P value |
| ARI^1^ | n=10 | n=65 |  | n=46^4^ | n=63 |  |
| Cough | 9 (90.0) | 58 (89.2) | 1.000 | 45 (97.8) | 62 (98.4) | 1.000 |
| Sore throat | 9 (90.0) | 41 (63.1) | 0.150 | 23 (50.0) | 29 (46) | 0.702 |
| Breathlessness | 1 (10.0) | 4 (6.2) | 0.521 | 23 (50.0) | 27 (42.9) | 0.560 |
| Running nose | 7 (70.0) | 34 (52.3) | 0.333 | 22 (47.8) | 33 (52.4) | 0.700 |
| T>=37.5^o^C | 7 (70.0) | 53 (81.5) | 0.408 | 26 (56.5) | 53 (84.1) | 0.002 |
| T>=37.8^o^C | 6 (60.0) | 44 (67.7) | 0.723 | 25 (54.3) | 51 (81) | 0.003 |
| T>=38^o^C | 6 (60.0) | 35 (53.8) | 1.000 | 24 (52.2) | 48 (76.2) | 0.014 |
| FRI^5^ | 8 (80.0) | 62 (95.4) | 0.129 | 40 (87.0) | 60 (95.2) | 0.163 |
| ILI-U^6^ | 6 (60.0) | 43 (66.2) | 0.731 | 24 (52.2) | 50 (79.4) | 0.003 |
| ILI-W^7^ | 6 (60.0) | 30 (46.2) | 0.506 | 20 (43.5) | 44 (69.8) | 0.010 |
| 1. ARI: episode with acute onset with any key respiratory symptoms including cough, shortness of breath, sore throat, or runny nose.  2. Historical-FLU, public health indications: ARI cases referred to TTSH for public health indications following clinical suspicion of having influenza A(H1N1)pdm09 based on epidemiological risk factors like travel and contact history  3. Historical-FLU, medical indications: patients with medical indications requiring hospitalisation for care and treatment  4. 5 episodes positive for more than 1 panel virus are excluded.  5. FRI: ARI with self-reported fever, regardless of body temperature (T) measurement  6. ILI-U: ILI as defined by Centers for Disease Control and Prevention of the United States of America, i.e. fever ≥37.8˚C together with cough and ⁄or sore throat in the absence of a known cause other than influenza  7. ILI-W: ILI as defined by World Health Organization, i.e. fever of ≥38˚C plus cough with onset within the last 10 days | | | | | | |

Sensitivity analysis on the clinical criteria and case definitions for detecting viral infection

In our main manuscript, we grouped all community-ARI episodes (none of which required hospitalisation) with historical-FLU admitted for public health indications as mild-ARI, and inpatient-ARI were grouped with historical-FLU cases admitted for medical indications as severe-ARI. This increased the number of influenza cases available for evaluating the discriminatory value of clinical parameters and case definitions. We performed a sensitivity analysis excluding the Historical-FLU cases and the results were essentially the same other than for the wider confidence intervals (Fig. S1).

**Figure. S1. Comparison of the ability of different case definitions and temperature cut-off points in discriminating influenza, other viral respiratory infections and ARIs negative on MRT-PCR.** Discriminating influenza from other causes of ARI in community-ARI (A) and inpatient-ARI (C), and discriminating infections positive for any MRT-PCR panel virus from samples negative by MRT-PCR for community-ARI (B) and inpatient-ARI (D). Figure is based only on results from mono-infection episodes and does not include additional positive results from the PathChip system.

***References***

1. Tay, J., Ng, Y. F., Cutter, J. L. & James, L. Influenza A (H1N1-2009) pandemic in Singapore--public health control measures implemented and lessons learnt. *Ann. Acad. Med. Singapore* **39,** 313–312 (2010).

2. Mukherjee, P. *et al.* Epidemiology of Travel-associated Pandemic (H1N1) 2009 Infection in 116 Patients, Singapore. *Emerg. Infect. Dis.* **16,** 21–26 (2010).
